# Heredity

**Published:** 1912-12

**Authors:** Theo. Y. Hull

**Affiliations:** Secretary Texas State Society of Social Hygiene, San Antonio, Texas


### Heredity.

BY THEO. Y. HULL, B. S., M. D., SECRETARY TEXAS STATE SOCIETY OF SOCIAL HYGIENE, SAN ANTONIO, TEXAS.

It is my purpose in this paper to state as simply as possible a few of the basic principles upon which the science of heredity rests, and to suggest their bearing upon the question of race culture, which, sooner or later, the very nature of our civilization will compel us to consider. While our conception of these principles is yet far from perfect, it is a conception far in advance of that of a generation ago. The possession of definite ideas of the importance of heredity at a time when the interest of the intellectual world is centered in matters relating to human welfare is certain to encourage study and to bring about at least a partial application of these principles.

Before definite information, old beliefs, sometimes wholly erroneous, sometimes half true and sometimes masking the truth, are dispelled. The very indefiniteness of our ideas found expression a couple of generations ago in the ridicule of Balzac, who defined heredity to be a maze in which science loses itself, and in the present generation, in the criticism of Bernard Shaw, who spoke of it as a bubble to be pricked.

But the lust of knowledge and the lure of the unknown impel investigation, with the result that science has found itself, and heredity is not a bubble. The spirit of research animated the soul of the abbot of Brunn, and within the confines of his convent walls the science of heredity was born. Unfortunately, the report of his theories, experiments and conclusions was announced at a time when the scientific world was engrossed in the controversy aroused by Darwin. With the announcement of similar discoveries by DeVries in 1900, the world awoke to the fact that the abbot's epoch-making work had lain hidden for a full third of a century. To Gregor Johann Mendel, abbot of Brunn, belongs the credit of first formulating the laws of heredity.

Mendel, with the persistency of the true scientist, pursued his experiments during a period of eight years before publishing his observations. His program was to discover the law of inheritance in hybrid plants. He selected for his experiments the edible pea. This plant fulfilled certain requirements: (a) It possessed constant differentiating characters; (b) it admitted of easy artificial pollination; (c) the hybrids were readily fertile; (d) they could be protected from foreign pollen.

The differentiating characters of the edible pea are seven. In some the vines are tall, while others are dwarf. The form of the ripe seed in some is round and smooth, while in others it is angular and wrinkled. Each of the characteristics, as tallness and dwarfness, was tested for constancy. The result of the experiments was the discovery of *the law of dominance* and *the law of segregation*. To prove the law of dominance, Mendel crossed plants having opposing characters. For instance, the pollen of a dwarf plant was transferred to the stigma of a tall plant. The same artificial pollination of plants with opposing characters was accomplished in each of the seven variations. In each case the hybrid plants grown from the seeds of the artificially pollinated flowers possessed only one of the opposing characters to the exclusion of the other. When the dwarf pea was crossed with the tall, the hybrid plants were all tall, no dwarfs and no intermediates appearing. This was found invariably true of each of the remaining six pairs of characters. The character that always prevailed was designated as the *dominant character*, and that which had receded or was apparently lost was called the *recessive character*. In the experiments tallness was a dominant, while dwarfness was a recessive, character. Mendel thus established his first law of domination by experiments on plants. But other observers have confirmed the law in other plants and animals. For instance, when the gray house mouse is crossed with a white mouse, the offspring of the first generation are all gray. The same is true af gray and white rabbits. Grayness is a dominant character, while albinism is a recessive.

While the first generation of hybrids seemed to possess only the dominant character, the next generation revealed the fact that the recessive character was not lost, for when the plants of the first hybrid generation were self-pollenized, the ripened seeds developed plants of both characters. Evidently the recessive character was present in the germ all along with the dominant character, but, for some reason, it was suppressed.

While the second generation established the fact that the recessive character is not lost, it established also the fact that both characters appeared in a certain ratio. The plants grown from the seed of the first self-fertilized hybrid numbered three dominants to one recessive. Out of 1064 plants of this second generation grown with reference to the length of the stem, 787 were tall and 277 dwarf. In all the other characters tested, the same splitting up or segregation occurred. The ratio of 3 to 1 held good in experiments upon mice (gray and albino), corn (field and sugar corn), silk worms (yellow and white cocoons), snails (five banded forms and bandless forms) and many other species of both plants and animals. An interesting illustration of this segregation is seen in the hybrids resulting from crossing Angora rabbits (long hair) with Belgian hares (short hair). The offspring of these hybrids, inbred, were of both varieties. Of seventy that reached the age of two months, 53 were short-haired and 17 long-haired.

Continuing the experiments with the seeds of the second generation of hybrid peas, it was found that the plant with the recessive character, dwarfness, self-fertilized, bred true for successive generations to the recessive character—all were dwarfs. On the other hand, when the three remaining plants, all possessing the dominant character, tallness, were self-pollenized, another peculiarity was observed—one plant gave rise to plants containing the dominant character only and continued to breed true through successive generations. The remaining two plants, under the same conditions, gave rise to plants of both varieties, always in the proportion of three dominants to one recessive.

These experiments proved that the fixed characters of the plants were always transmitted in the proportion of one pure recessive—having only this character—one pure dominant, and two impure dominants, having both characters, with the recessive suppressed.

If we assume the establishment of Mendel's laws of dominance and segregation, where and how do these changes occur?

Heredity, strictly speaking, has only to do with the germ-cells and the characters which they unite in the new organism. Whatever befalls the new organism after the moment of fusion of the germ-cells is not heredity, but is due to its environment and its food supply. At the moment of the fusion of the parental germ-cells, the destiny of the new organism, whether it be plant or animal, is sealed in so far as its parental characters are concerned. Weissmann holds that the germinal matter is continuous from generation to generation. If this be true, the pairs of differentiating characters are passed along, under one set of conditions the dominant character predominating, and under another the recessive.

Whenever the pollen granule falls upon the receptive stigma, a pollen tube develops, which penetrates the tissues of the stigma and style and enters the germ-sac of the ovule, uniting the substance of the pollen-cell with that of the egg-cell. A fusion of the nuclei of the two germ-cells follows. The germ-cells carry with them and unite in the new cell the characteristics of both parents and the manner of their union determines the traits of the new organism. The pollen granule of the tall plant falling upon the stigma of the dwarf will unite within the new germ-cell the characters of both.

At the moment of fusion of the nuclei of the two cells, those changes occur which are manifest in the dominance of one differentiating character over its opposing character, and also in the order of their appearance. Whether these changes are chemical or physical we know not. We can only judge by comparing certain outward manifestations. In sweet corn the grain is angular and shriveled, while in the field variety it is rounded and full. The second character is dominant to the first. The reserve material in the rounded grain is largely starch, while in the shriveled grain it is largely sugar from which the water has been abstracted. The conversion of the glucose into starch in the full grains is more complete than in the shriveled. The absorption of the excess of water in the latter leaves the grain shriveled. This suggests a ferment which was present in the one and absent or partially absent in the other. While we do not fully understand the chemistry of color in plants and animals, it is possible to conceive a ferment acting upon a chromogen factor. In the case of the mice referred to earlier, albinism is recessive to grayness. In the albino we can easily understand the absence of a ferment. This phase of the question presents many problems which require deeper study for their solution.

In the foregoing I have endeavored to present the basic principles of heredity as we have observed them in the lower organism, both plant and animal. There is no reason to believe that the processes are different in the higher organisms, although manifestly more complex. In man there are many obstacles that prevent their strict application. One obstacle is an almost universal misconception of what constitutes inheritance, and attributing to it many things that are entirely due to environment and nutritional conditions. It matters little whether these conditions are prenatal or post-natal—they operate in precisely the same manner.

The idea of the inheritance of disease is almost as old as civilization itself. Ignorance of the true cause of many diseases lead to their being ascribed to heredity, and we are still ascribing to heredity conditions that are undoubtedly due to disordered metabolism. Sir William Church, in discussing the subject of heredity and disease before the Royal Society of Medicine, said: "No disease which arises from or is associated with the presence of a foreign body, whether living or dead, within us can be considered hereditary." The diseases tuberculosis and syphilis, so universally ascribed to heredity a generation ago, we know to be due to specific bacteria. The micro-organisms within the body are foreign bodies, and for these diseases to be inherited they must be transmitted with the germ-cells, which is practically impossible. We may assume, with little fear of contradiction, that no disease per se is inherited.

But, on the other hand, we are all familiar with the occurrence of certain disorders in families for several generations. The natural inference in such cases is that something else is handed down from parent to offspring that renders them either more liable to, or less able to resist, the parental affection. Personally, I believe the conditions so frequently spoken of as predisposition can be explained by two facts—the greater exposure to infection early in life, possibly pre-natal, and to disturbed metabolism, either before or after fusion of the germ-cells. We must not forget that the germ-cell is a living entity that draws its nourishment from the nutrient fluids of the body in which it exists. If these fluids be filled with toxic substances, bacterial poisons, etc., capable of affecting the germ-cell, its development will be retarded or disturbed, and this effect may be manifest for several generations. Whether this be the true explanation of predisposition, or whether the "inborn errors of metabolism" of Garrod be due to the absence of special ferments in some families which are present in others, is a question yet to be solved. The explanation of albinism and the shriveled grains of corn lends some probability to this theory. But this part of the problem remains to be worked out. It includes the possibility of the transmissibility of acquired characteristics, which is denied by many. But no matter whether predisposition is due to the presence or absence of something, or is the result of a faulty development, it remains a factor that we must bear in mind in our study of disease.

Eliminating the stigmata of degeneracy, which, in my opinion, are due to toxic and nutritional disturbances of the germ-cell either before or after fusion, and which may be operative for many generations, the characters which may be transmitted are physical, mental and moral.

Mendel's discoveries lead us to look upon our own inheritance as made up of unit-characters. These unit-characters, whether good or ill, do not disappear, but reapper persistently in their original purity. Mr. Punnett says: "Permanent progress is a question of breeding rather than of pedagogics; a matter of gametes, not training. As our knowledge of heredity clears, and the mists of superstition are dispelled, there grows upon us with an ever-increasing and relentless force the conviction that the creature is not made, but born." Bateson says: "Education, sanitation and the rest are but the giving or withholding of opportunity." According to this view, our physical, mental and moral characters are inherent in us and that our later training is simply developmental. This is the latest conception of education.

We are now on the threshold, undoubtedly, of important race studies which we seemingly fear to take up. Can we apply Mendelian principles to man? The recent origin of the scientific study of heredity in man, the length of a single generation, and the want of reliable data covering several generations, are all serious obstacles. Thompson says: "We cannot but feel that the application of biological results is only beginning." There is no reason to suppose that the human family occupies a place in nature separate and distinct from other creatures, or that it is governed by different biological laws; but the higher the organism, the more complex and varied the results. Bateson says: "There are others who look to the science of heredity with a loftier aspiration; who ask, can any of this be used to help those who come after to be better than we are—healthier, wiser, or more worthy?" Our present knowledge suggests the possibility. It is much a question of whether sentiment or intelligence shall be the selective agent.

We have now what might very appropriately be termed microbic selection. Our fearful and largely unnecessary death rate from infectious diseases thins our ranks in a very indiscriminate manner, and some would have us believe that this is evolution. Are those who escape stronger, or more fortunate? If this kind of selection is helpful, the race should have reached an exalted state of immunity centuries ago. Could intelligent selection, though admittedly unpopular, be more harmful? If unrestrained, which will grow the more rapidly in the garden—weeds or flowers? Pearson points out that 25 per cent of the married couples in England produce 50 per cent of the next generation. Does nothing depend upon the physical, the mental and the moral condition of this 25 per cent? Statistics show that the ratio of defectives increased between the years 1874 to 1896 from 5.4 per 1000 over 15 years of age to 11.6. Does this look like intelligent selection, or that there is no need for a practical eugenics? Emerson said: "We are breeding men with too much guano in their composition." The statistics of racial degeneration are based on grim facts.

Whether we consider the Mendelian principles of inheritance to apply to human selection or not, we cannot deny that intelligent selection does apply, and this is the great question after all. How can we arouse people to think of these matters? To the future generation it means much. Has not every child a right to be well born? Certain it is that if we are to maintain the individual efficiency, we, as a people, must give thought to these matters. The most important factor in human progress is human selection.

